# Prevalence, progress, and social inequalities of home deliveries in Ghana from 2006 to 2018: insights from the multiple indicator cluster surveys

**DOI:** 10.1186/s12884-021-03989-x

**Published:** 2021-07-21

**Authors:** Veronica Millicent Dzomeku, Precious Adade Duodu, Joshua Okyere, Livingstone Aduse-Poku, Nutifafa Eugene Yaw Dey, Adwoa Bemah Boamah Mensah, Emmanuel Kweku Nakua, Pascal Agbadi, Jerry John Nutor

**Affiliations:** 1 Department of Nursing, Faculty of Allied Health Sciences, College of Health Sciences, Kwame Nkrumah University of Science and Technology, Kumasi, Ghana; 2 Department of Nursing and Midwifery, School of Human and Health Sciences, University of Huddersfield, Queensgate Huddersfield, England, UK; 3 Department of Population and Health, University of Cape Coast, Private Mail Bag, Cape Coast, Ghana; 4 Department of Epidemiology, College of Public Health & Health Professions, College of Medicine, University of Florida, Florida, USA; 5 Department of Psychology, University of Ghana, P.O. Box LG 84, Legon, Ghana; 6 Department of Epidemiology and Biostatistics, School of Public Health, Kwame Nkrumah University of Science and Technology, Kumasi, Ghana; 7 Department of Family Health Care Nursing, School of Nursing, University of California San Francisco, San Francisco, CA USA

**Keywords:** Prenatal care, Antenatal care, Pregnancy, Skilled birth attendance

## Abstract

**Background:**

Delivery in unsafe and unsupervised conditions is common in developing countries including Ghana. Over the years, the Government of Ghana has attempted to improve maternal and child healthcare services including the reduction of home deliveries through programs such as fee waiver for delivery in 2003, abolishment of delivery care cost in 2005, and the introduction of the National Health Insurance Scheme in 2005. Though these efforts have yielded some results, home delivery is still an issue of great concern in Ghana. Therefore, the aim of the present study was to identify the risk factors that are consistently associated with home deliveries in Ghana between 2006 and 2017–18.

**Methods:**

The study relied on datasets from three waves (2006, 2011, and 2017–18) of the Ghana Multiple Indicator Cluster surveys (GMICS). Summary statistics were used to describe the sample. The survey design of the GMICS was accounted for using the ‘svyset’ command in STATA-14 before the association tests. Robust Poisson regression was used to estimate the relationship between sociodemographic factors and home deliveries in Ghana in both bivariate and multivariable models.

**Results:**

The proportion of women who give birth at home during the period under consideration has decreased. The proportion of home deliveries has reduced from 50.56% in 2006 to 21.37% in 2017–18. In the multivariable model, women who had less than eight antenatal care visits, as well as those who dwelt in households with decreasing wealth, rural areas of residence, were consistently at risk of delivering in the home throughout the three data waves. Residing in the Upper East region was associated with a lower likelihood of delivering at home.

**Conclusion:**

Policies should target the at-risk-women to achieve complete reduction in home deliveries. Access to facility-based deliveries should be expanded to ensure that the expansion measures are pro-poor, pro-rural, and pro-uneducated. Innovative measures such as mobile antenatal care programs should be organized in every community in the population segments that were consistently choosing home deliveries over facility-based deliveries.

## Introduction

Improving the maternal health of women is essential to their overall health and wellbeing. Evidence shows that about 140 million women give birth per annum, with about 810 to 890 dying daily as a result of preventable causes related to pregnancy and childbirth [1, 2], and sub-Saharan Africa (SSA) alone accounts for about two-thirds of all these maternal deaths [3]. Consequently, several efforts have been coordinated by international organizations and individual governments to significantly reduce maternal mortality to under 70 deaths per 100,000 live births by 2030, as a global target set by the Sustainable Development Goal three (SDG 3.1) [2, 4]. One of such efforts has been the global campaign to reduce home deliveries and increase institutional birth deliveries (IBD) towards skilled birth attendance (SBA), a critical benchmark indicator for monitoring the progress of the Millennium Development Goal five (MDG 5) as well as the new SDG 3 and with a 90% global target [4]. This has seen an increase in IBD from as low as 5% in 2005 to 48% in 2019 [1]. Home delivery refers to the practice of childbirth that occurs at the place of residence of the pregnant woman or the homes of other people [5, 6]. These births are attended by unskilled personnel including traditional birth attendants (TBAs), relatives, and friends as substitutes for skilled birth attendants [5, 7]. A skilled birth attendant is “an accredited health professional – such as a midwife, doctor or nurse – who has been educated and trained to proficiency in the skills needed to manage normal (uncomplicated) pregnancies, childbirth, and the immediate postnatal period, and in the identification, management and referral of complications in women and newborns” [8]. Home delivery continues to gain considerable attention due to its strong association with higher neonatal and maternal mortalities arising from concomitant obstetric complications such as sepsis, peripartum cardiomyopathy, embolism, haemorrhage, as well as other obstetric dangers [9–11]. Other reported risks for home delivery include abandonment of the recommended practices of colostrum provision and breastfeeding, deserting the child and mother’s immunizations and nutrition supplementation, and lack of check-up for the child and mother postnatally [12, 13].

Ghana, an SSA country, has a national SBA target of 80% towards shifting the paradigm from home deliveries to IBD [14]. In 2016, the proportion of births or deliveries by skilled birth attendants was 56.2%, and this was significantly below the national target [14]. The national SBA target is part of the maternal and child health interventions to reduce Ghana’s maternal mortality ratio (MMR) which currently stands at 310 per 100,000 live births and are largely attributable to inadequate access to quality skilled delivery, emergency obstetric, and newborn care and family planning [15]. Ghana’s MMR is still very high compared to the global target of less than 70 per 100,000 live births by 2030 [4], and therefore the implementation of policy interventions including an improved shift from home deliveries to IBD is critical. Reports on Ghana’s regional trend in skilled delivery from 2014 to 2016 indicate that Upper East Region and Volta Region consistently recorded the highest and lowest skilled delivery coverage over the 3 years, respectively [14]. Ghana has made substantial progress to reduce the prevalence of home deliveries by reducing some social inequalities through the introduction of the Community-based Health Planning and Services (CHPS) initiative, and the free maternal health care policy through the National Health Insurance Scheme (NHIS) in 2005 [16, 17]. These initiatives contributed significantly to reducing home delivery from 45% in 2007 to 20% in 2017 [18]. Despite the significant concerted global and national efforts, uptake of IBD remains low, with many childbearing women continuing to deliver their babies at home due to limited access to maternal healthcare services including delivery services [7, 19, 20]. Hence, there is a need to estimate the prevalence and explore the nuances associated with home delivery.

Available global evidence indicates that women’s decisions to deliver at home or at a health institution is influenced by prevailing social inequalities, and these substantial inequalities have the greatest burden among the poor and lower social strata [21, 22]. Social inequalities manifesting in the dimensions of education, wealth quintile, place of residence, distance to the health facility, among others have been posited to influence the prevalence and progress to reduce home deliveries [23]. In Ghana, previous studies report that social inequalities such as rural residency, poor wealth status, no formal education, not being covered by the national health insurance scheme, and male-headed households exacerbate home deliveries in the country [5, 24–26]. However, to the best of our knowledge, no study has investigated the progress that has been made in reducing home delivery over the years as well as examine the magnitude of the social inequalities relating to home delivery in Ghana. Therefore, this study sought to fill the dearth in literature by examining the prevalence, progress and social inequalities associated with home deliveries from 2006 to 2018 using data from the Multiple Indicator Cluster Survey (MICS). The study findings will inform policy interventions towards attaining both the national and global targets regarding maternal and child health.

## Methods

### Data and collection procedure

The current study used datasets collected in three waves by the Ghana Multiple Indicator Cluster Survey (GMICS) in 2006, 2011, and 2017/2018. The GMICS is a cross-sectional survey conducted by Ghana Statistical Service (GSS) in collaboration with the Ghana Health Service (GHS), Ministry of Health (MOH), and the Ministry of Education [27]. The survey received funding and technical support from the United Nations International Children's Emergency Fund (UNICEF) and other international donors [27]. The primary goal of the MICS surveys is to analyze key indicators that assist countries to produce data for use in national development plans, policies, and programmes. On top of that, the GMICS is intended to assess progress towards SDGs and other agreements signed internationally [27].

MICS surveys use a multi-stage stratified cluster design to select a probability sample of households and women (15–49 years). This approach was used to nationally survey women in urban and rural areas from the erstwhile ten administrative regions in Ghana namely, Western, Central, Greater Accra, Volta, Eastern, Ashanti, Brong Ahafo, Northern, Upper East, and Upper West. At the first stage based on the 2010 Population and Housing Census (PHC) of Ghana, enumeration areas (EAs) were randomly selected, becoming the primary sampling units (PSUs). Every household within the EA is listed to create a sampling frame and a sample of households was chosen in the second stage using systematic random sampling. Then reproductive-aged women were recruited from these selected households. A total of 7,795 women within the ages of 15 to 49 years who had delivered two years before the data collection for all the three waves participated in this study.

### Measures

#### Outcome variable

The outcome variable is the place of delivery, therefore home delivery is the focal point for the present study. This variable was derived from the survey question asking the participants about the place of their child delivery two years before the start of the survey. Participants were specifically asked this question, “Where did you give birth to (name of child)?” The response format to this question were these: Home (“respondent’s home” and “other’s home”); Public medical sector (“Government hospital”, “Government clinic/health centre”, “Government health post” and “Other public”); Private medical sector (“Private hospital”, “Private Clinic”, “Private maternity home” and “Other private medical”); and Other. We assigned a value of “1” to the home response and all other options were assigned “0”.

#### Explanatory variables

The explanatory variables in the models were selected after a review of the literature and their availability in the dataset [28–30]. The authors explored the following variables: age of woman, education, polygyny, wanted last-child, parity, antenatal care (ANC) attendance, previous child loss experience, health insurance, household wealth index, urban–rural residence, and region of residence. The ANC variable was recoded as 0–3 times (less than 3), 4–7 times, and 8 times and above. It would have been helpful to compare women who did not attend ANC at all with the other categories, but data on ANC attendance in 2006 revealed that only one woman indicated she did not have an ANC visit. Therefore, to make ANC effect on Home delivery comparable over time, we decided to group those with no ANC attendance with those who had 1 up to 3 visits. We included the variable on respondent’s previous child loss experience in our models to ascertain its association with giving birth to their children in the home. It is not clear from the dataset or the questionnaire whether the experience of child loss occurred in a health facility or the home or any other place.

All these variables were available in all three datasets except that of health insurance which was available in 2011 and 2017/2018; we included this variable because of its policy implication on maternal and child health. We did not include in our model the variable on religious affiliations of the respondents because it had no data on it in the most recent GMICS dataset (the 2017/18 dataset). As indicated in Table 1, participants responded to all the variables using simple response options.

**Table 1:** Summary statistics of sociodemographic correlates and home deliveries in Ghana, 2006 to 2017–18

	2006			2011			2017–18		
	*Delivered at home*			*Delivered at home*			*Delivered at home*		
	n (%)	NO	YES	N	NO	YES	n%	NO	YES
**Total**	**1456 (100)**	**49.44**	**50.56**	**2873 (100)**	**68.65**	**31.35**	**3466 (100)**	**78.63**	**21.37**
**Age (years)**	***P *= 0.560**			***P *≤ 0.005**			***P *= 0.300**		
15–24	433 (29.73)	47.72	52.28	705 (24.53)	69.35	30.65	952 (27.47)	78.36	21.64
25–34	693 (47.60)	51.45	48.55	1436 (49.97)	71.60	28.40	1634 (47.16)	79.10	20.90
35 +	330 (22.67)	47.45	52.55	733 (25.50)	62.18	37.35	879 (25.37)	78.03	21.97
**Education**	***P *≤ 0.001**			***P *≤ 0.001**			***P *≤ 0.001**		
None or pre-primary	537 (36.87)	30.76	69.24	833 (29.00)	43.48	56.52	774 (22.34)	67.24	32.76
Primary	320 (21.98)	46.56	53.44	642 (22.34)	67.24	32.76	729 (21.02)	72.97	27.03
JHS	496 (34.05)	63.99	36.01	1007 (35.04)	80.01	19.99	1341 (38.70)	81.02	18.98
Secondary and above	103 (7.09)	85.58	14.42	391 (13.62)	95.33	4.67	622 (17.94)	94.28	5.72
**Polygyny**	***P *≤ 0.001**			***P *≤ 0.001**			***P *≤ 0.001**		
Never/ formerly married	165 (11.33)	59.24	40.76	293 (10.19)	75.05	24.95	592 (17.08)	77.61	22.39
In one union	1,027 (70.52)	51.36	48.64	2112 (73.52)	72.01	27.99	2331 (67.25)	81.27	18.73
Have co-wives	264 (18.14)	35.85	64.15	468 (16.29)	49.47	50.53	543 (15.66)	68.37	31.63
**Wanted last-child**	***P *= 0.060**			***P *= 0.107**			***P *= 0.109**		
Yes	884 (60.74)	51.34	48.66	1630 (56.74)	69.30	30.70	1711 (49.37)	79.75	20.25
Later/No More/others	572 (39.26)	46.50	53.50	1243 (43.26)	67.79	32.21	1755 (50.63)	77.53	22.47
**Parity**	***P *≤ 0.001**			***P *≤ 0.001**			***P *≤ 0.001**		
Primiparous	321 (22.04)	62.47	37.53	619 (21.54)	86.73	13.27	791 (22.82)	84.60	15.40
Double	301 (20.65)	55.34	44.66	527 (18.33)	72.72	27.28	660 (19.05)	81.63	18.37
Multiparous	834 (57.30)	42.30	57.70	1727 (60.13)	60.93	39.07	2015 (58.13)	75.29	24.71
**ANC attendance**	***P *≤ 0.001**			***P *≤ 0.001**			***P *≤ 0.001**		
less than 4 times	389 (26.71)	25.26	74.74	384 (13.38)	32.89	67.11	519 (14.98)	51.66	48.37
4–7 times	717 (49.23)	50.75	49.25	1436 (49.98)	66.55	33.45	2031 (58.61)	80.60	19.40
8 times and more	350 (24.06)	73.58	26.42	1053 (36.64)	84.57	15.43	915 (26.41)	89.55	10.45
Previous child loss experience	***P *≤ 0.001**			***P *≤ 0.001**			***P *≤ 0.001**		
No	1085 (74.51)	52.09	47.91	2186 (76.08)	72.07	27.93	2849 (82.20)	79.85	20.15
Yes	371 (25.49)	41.68	58.32	687 (23.92)	57.75	42.25	617 (17.80)	72.99	27.01
**Health Insurance**				***P *≤ 0.001**			***P *≤ 0.001**		
Uninsured	—	—	—	773 (26.89)	55.51	44.49	1311 (37.82)	69.11	30.89
Insured	—	—	—	2100 (73.11)	73.48	26.52	2155 (62.18)	84.41	15.59
**Household wealth**	***P *≤ 0.001**			***P *≤ 0.001**			***P *≤ 0.001**		
Poorest	335 (23.00)	22.88	77.12	637 (22.16)	38.92	61.08	747 (21.55)	63.17	36.83
Poorer	347 (23.86)	32.34	67.66	621 (21.61)	58.68	41.32	694 (20.03)	70.95	29.05
Middle	277 (19.04)	45.78	54.22	568 (19.79)	70.60	29.40	676 (19.51)	77.67	22.33
Richer	286 (19.61)	74.70	25.30	517 (17.98)	85.53	14.47	709 (20.45)	87.04	12.96
Richest	211 (14.50)	90.33	9.67	530 (18.46)	97.48	2.52	640 (18.46)	96.69	3.31
**Urban–Rural residence**	***P *≤ 0.001**			***P *≤ 0.001**			***P *≤ 0.001**		
Urban	498 (34.21)	77.76	22.24	1214 (42.25)	88.01	11.99	1464 (42.25)	90.17	9.83
Rural	958 (65.79)	34.71	65.29	1659 (57.75)	54.48	45.52	2002 (57.75)	70.18	29.82
**Region**	***P *≤ 0.001**			***P *≤ 0.001**			***P *≤ 0.001**		
Western	154 (10.60)	39.41	60.59	306 (10.66)	63.15	36.85	400 (11.53)	78.98	21.02
Central	112 (7.70)	47.25	52.75	279 (9.72)	63.81	36.19	341 (9.83)	74.75	25.25
Greater Accra	177 (12.16)	83.70	16.30	451 (15.71)	89.16	10.84	332 (9.58)	93.21	6.79
Volta	103 (7.10)	43.55	**58.48**	214 (7.46)	64.35	**35.65**	285 (8.23)	68.59	**31.41**
Eastern	195 (13.37)	41.52	58.48	327 (11.37)	78.58	21.42	402 (11.60)	78.56	21.44
Ashanti	222 (15.22)	59.65	40.35	511 (17.78)	75.78	24.22	788 (22.73)	81.72	18.28
Brong Ahafo	115 (7.87)	57.23	42.77	258 (8.99)	62.10	37.90	330 (9.51)	86.64	13.36
Northern	278 (19.10)	34.62	**65.38**	321 (11.17)	38.51	**61.49**	388 (11.19)	58.33	**41.67**
Upper East	61 (4.16)	43.60	56.40	120 (4.17)	66.45	33.55	112 (3.25)	94.93	5.07
Upper West	40 (2.72)	29.08	70.92	85 (2.97)	62.13	37.87	88 (2.55)	80.77	19.23

### Data preparation and analysis

The datasets were cleaned, and variables recoded in STATA version 14. We accounted for survey weights for the differential probability selection of the sample. The variances were calculated to adjust for clustering, stratification, and design effects using the Taylor linearization technique [31]. We first conducted specific survey waves (2006, 2011, and 2017–18) univariates analyses, computing frequencies and percentages of all variables (Table 1—second, fifth, and eighth columns). Secondly, bivariate analyses were performed with Chi-square test of independence, estimating the relationship between the explanatory variables and outcome variable (place of delivery – home or facility delivery) as presented in Table 1. Lastly, multivariate analyses with robust Poisson regression models incorporating all explanatory variables were used to model the prevalence of home delivery as well as examine its relationship, regardless of statistical significance in the bivariate analyses as presented in Table 2. Because Poisson regression is applied to a binary variable, the robust error variance technique is used to avoid overestimating the error of the estimated prevalence ratio (PR). The preference for prevalence ratio over odds ratio is adequately explained elsewhere [32, 33], and the same thing applies to our study. The prevalence ratio and the adjusted prevalence ratio are reported.

**Table 2:** Sociodemographic correlates regressed on home deliveries in Ghana, 2006 to 2017–2018

	2006		2011		2017–18	
	PR [95% CI]	APR [95% CI]	PR [95% CI]	APR [95% CI]	PR [95% CI]	APR [95% CI]
**Total**						
**Age (years)**						
15–24	0.995 [0.843,1.174]	1.242* [1.038,1.486]	0.810* [0.660,0.994]	1.333* [1.054,1.685]	0.985 [0.774,1.253]	1.036 [0.774,1.386]
25–34	0.924 [0.796,1.072]	1.100 [0.976,1.238]	0.751*** [0.633,0.890]	1.040 [0.893,1.211]	0.951 [0.764,1.183]	1.112 [0.900,1.374]
35 +	Ref	Ref	Ref	Ref	Ref	Ref
**Education**						
None or pre-primary	4.802*** [2.644,8.723]	1.677 [0.993,2.832]	12.11*** [6.998,20.95]	2.131** [1.216,3.736]	5.724*** [3.465,9.457]	1.786* [1.070,2.982]
Primary	3.706*** [2.032,6.759]	1.462 [0.868,2.463]	7.018*** [4.006,12.30]	1.671 [0.960,2.908]	4.724*** [2.852,7.824]	1.782* [1.061,2.994]
JHS	2.498** [1.379,4.525]	1.251 [0.759,2.061]	4.284*** [2.411,7.609]	1.461 [0.830,2.573]	3.317*** [2.075,5.303]	1.705* [1.039,2.800]
Secondary and above	Ref	Ref	Ref	Ref	Ref	Ref
**Polygyny**						
Never/formerly married	Ref	Ref	Ref	Ref	Ref	Ref
In one union	1.193 [0.937,1.520]	1.119 [0.908,1.379]	1.122 [0.841,1.497]	0.876 [0.686,1.117]	0.837 [0.645,1.086]	0.889 [0.670,1.180]
Have co-wives	1.574*** [1.209,2.049]	1.201 [0.942,1.531]	2.025*** [1.488,2.756]	0.943 [0.715,1.244]	1.413* [1.032,1.934]	1.051 [0.758,1.457]
**Wanted last child**						
Yes	Ref	Ref	Ref	Ref	Ref	Ref
Later/No More/others	1.099 [0.966,1.252]	1.042 [0.932,1.166]	1.049 [0.904,1.218]	1.119 [0.975,1.285]	1.109 [0.935,1.316]	1.045 [0.886,1.232]
**Parity**						
Primiparous	Ref	Ref	Ref	Ref	Ref	Ref
Double	1.190 [0.960,1.475]	1.337** [1.103,1.621]	2.056*** [1.526,2.771]	1.832*** [1.384,2.426]	1.193 [0.900,1.582]	1.242 [0.941,1.641]
Multiparous	1.537*** [1.279,1.848]	1.398** [1.139,1.717]	2.945*** [2.263,3.833]	2.263*** [1.684,3.042]	1.604*** [1.239,2.078]	1.231 [0.907,1.670]
**ANC attendance**						
less than 4 times	2.829*** [2.226,3.594]	1.605*** [1.322,1.950]	4.349*** [3.472,5.447]	1.767*** [1.412,2.211]	4.626*** [3.480,6.149]	2.443*** [1.808,3.301]
4–7 times	1.864*** [1.466,2.370]	1.291** [1.077,1.547]	2.167*** [1.753,2.679]	1.294* [1.057,1.583]	1.857*** [1.418,2.430]	1.302* [1.001,1.692]
8 times and more	Ref	Ref	Ref	Ref	Ref	Ref
Previous child loss experience						
No	Ref	Ref	Ref	Ref	Ref	Ref
Yes	1.217*** [1.091,1.359]	0.936 [0.841,1.040]	1.513*** [1.302,1.758]	0.957 [0.830,1.104]	1.340* [1.055,1.702]	1.049 [0.864,1.272]
**Health Insurance**						
Uninsured	—	—	1.678*** [1.430,1.968]	1.161* [1.017,1.325]	1.981*** [1.643,2.390]	1.517*** [1.283,1.794]
Insured	—	—	Ref	Ref	Ref	Ref
**Household wealth**						
Poorest	7.974*** [4.873,13.05]	3.441*** [1.967,6.016]	24.26*** [10.24,57.46]	6.689*** [2.376,18.83]	11.12*** [6.076,20.34]	4.240*** [2.248,7.999]
Poorer	6.997*** [4.240,11.55]	3.254*** [1.858,5.700]	16.41*** [6.826,39.44]	5.703*** [2.060,15.79]	8.768*** [4.810,15.98]	3.617*** [1.950,6.707]
Middle	5.606*** [3.329,9.439]	3.077*** [1.742,5.434]	11.67*** [4.789,28.46]	5.505** [1.995,15.19]	6.740*** [3.671,12.37]	3.222*** [1.697,6.118]
Richer	2.616*** [1.506,4.545]	1.908* [1.097,3.319]	5.747*** [2.273,14.53]	3.303* [1.217,8.962]	3.913*** [2.061,7.428]	2.509** [1.298,4.850]
Richest	Ref	Ref	Ref	Ref	Ref	Ref
**Urban–Rural residence**						
Urban	Ref	Ref	Ref	Ref	Ref	Ref
Rural	2.935*** [2.252,3.826]	1.504** [1.174,1.928]	3.796*** [2.939,4.903]	1.846*** [1.428,2.387]	3.033*** [2.137,4.305]	1.670** [1.205,2.314]
**Region**						
Western	3.717*** [2.162,6.388]	1.491 [0.984,2.261]	3.401*** [1.803,6.414]	0.940 [0.619,1.428]	3.094** [1.482,6.460]	1.683 [0.756,3.750]
Central	3.236*** [1.873,5.590]	1.336 [0.811,2.201]	3.339*** [1.794,6.217]	1.186 [0.821,1.715]	3.717*** [1.790,7.717]	2.040 [0.922,4.515]
Greater Accra	Ref	Ref	Ref	Ref	Ref	Ref
Volta	3.462*** [2.016,5.947]	1.198 [0.781,1.839]	3.290*** [1.693,6.392]	0.958 [0.609,1.508]	4.625*** [2.277,9.393]	1.699 [0.778,3.711]
Eastern	3.587*** [2.118,6.076]	1.307 [0.856,1.995]	1.976 [0.977,3.999]	0.836 [0.517,1.352]	3.157** [1.528,6.523]	1.489 [0.667,3.322]
Ashanti	2.475** [1.401,4.373]	1.244 [0.803,1.926]	2.235* [1.175,4.250]	0.851 [0.553,1.311]	2.691* [1.249,5.801]	1.712 [0.750,3.905]
Brong Ahafo	2.624** [1.439,4.783]	1.088 [0.681,1.736]	3.498*** [1.831,6.683]	0.908 [0.616,1.337]	1.968 [0.867,4.464]	0.984 [0.419,2.309]
Northern	4.010*** [2.316,6.945]	1.232 [0.795,1.908]	5.674*** [3.109,10.36]	1.094 [0.751,1.593]	6.135*** [3.065,12.28]	2.087 [0.948,4.597]
Upper East	3.459*** [1.973,6.066]	1.143 [0.731,1.787]	3.096*** [1.650,5.807]	0.633* [0.419,0.958]	0.747 [0.311,1.792]	0.329* [0.125,0.865]
Upper West	4.350*** [2.568,7.371]	1.273 [0.823,1.970]	3.494*** [1.872,6.522]	0.746 [0.498,1.118]	2.831** [1.326,6.044]	1.022 [0.440,2.373]
**Model details**						
Number of strata		20		20		20
Number of Primary Sampling Unit		291		775		649
Number of Observations		1456		2873		3466

Exponentiated coefficients; 95% confidence intervals in brackets

^*^p < 0.05, ** *p *< 0.01, *** *p *< 0.001

$$PR=\frac{OR}{\left(1+{p}_{1}*\left[OR-1\right]\right)}$$

where p_1_ is the prevalence of delivery at home.

We repeated these processes for all the three survey waves used in this study. Statistical significance is determined using 95% confidence intervals (CIs) and an alpha value of 0.05.

### Ethics approval and data availability

The study was performed in accordance with the Declaration of Helsinki and approved by appropriate ethics committee. Trained field enumerators collected data on behalf of UNICEF and GSS. The MICS team of UNICEF-Ghana, the Ethical Review Board of the Ghana Health Service, and the Ghana Statistical Service approved the study. Informed consent was obtained from all respondents, and assent was obtained for respondents younger than eighteen from parents/guardians/adult household member before data collection. More details regarding the data and ethical standards are available at: https://mics.unicef.org/surveys. Therefore, ethics approval for this study was not required since the data is secondary and is available in the public domain.

## Results

### Summary statistics of sociodemographic correlates and home deliveries in Ghana, 2006 to 2017–18

**Fig. 1:**
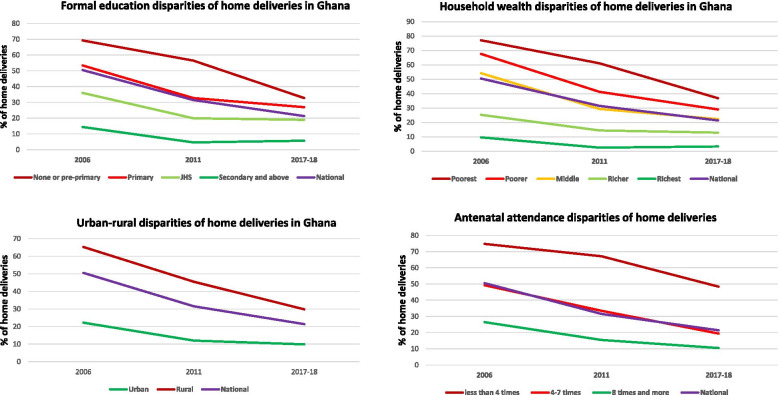
Trend graphs of sociodemographic disparities in home deliveries in Ghana, 2006 to 2017–18. Trend graphs showing consistent correlates of home deliveries in Ghana, 2006–2017-18

Generally, the proportion of women who give birth at home has decreased. The proportion of home deliveries has reduced from 50.56% in 2006 to 21.37% in 2017–18 (Table 1). The following sociodemographic factors were consistently associated with home deliveries in Ghana at a significant threshold of *P *≤ 0.001: education, polygyny, parity, antenatal care (ANC) attendance, previous child loss experience, health insurance, household wealth, urban–rural residence, and region of residence (Table 1). The proportion of childbearing women who gave birth to their children in their homes in these disadvantaged population segments (rural, poor households, none or low formal education) was consistently higher than the national average and their colleagues in advantaged population groups (Table 1): residing in a rural area [65.29% in 2006; 45.52% in 2011; 29.82% in 2017] (Fig. 1), residing in the poorest household [77.1 2% in 2006; 61.08% in 2011; 36.83% in 2017] (Fig. 1), and women without formal education [69.24% in 2006;56.52% in 2011; 32.76% in 2017] (Fig. 1). Of the wome who gave birth to children at home, higher proportion of them had the following sociodemographic characteristics: women who had co-wives [64.15% in 2006; 50.53% in 2011; 31.63% in 2017], women who had three or more children [57.70% in 2006; 39.07% in 2011; 24.71% in 2017], less than 4 times ANC attendance [74.74% in 2006; 67.11% in 2011; 48.37% in 2017] (Fig. 1), women who have ever had child loss expereince before their recent child [58.32% in 2006; 42.25% in 2011; 27.01% in 2017], women without health insurance [44.49% in 2011; 30.89% in 2017], and residing in the Volta [58.48% in 2006; 35.65% in 2011; 31.41% in 2017] and Northern [65.38% in 2006; 61.49% in 2011; 41.67% in 2017] regions.

### Sociodemographic correlates regressed on home deliveries in Ghana, 2006 to 2017–2018

In the multivariable model, ANC attendance, household wealth, urban–rural residence, and region of residence were consistently associated with home deliveries throughout the three data waves (Table 2; Fig. 1). We also highlighted the policy relevance of factors (such as formal education and having health insurance) that were significantly associated with home deliveries in the last two recent data waves (Table 2). Beyond indicating the consistent risk factors of home deliveries, we interpreted the significantly adjusted prevalence ratios of the 2017–18 dataset given that it represents current risk factors of home deliveries in Ghana (Table 2). Women with no education or pre-primary education, primary and junior high school education were more likely to deliver at home compared those with secondary school or higher education. Generally, there is a decreasing trend of delivery at home with increasing level of education. Also, women who attended ANC 4–7 times, or less than 4 times were more likely to deliver at home compared with those with 8 or more ANC attendance, Furthermore, women without health insurance subscription (compared to health insured women) and women in the richer or middle or poorer or poorest households (compared to women in the richest households) were more likely to deliver at home. Finally, women in rural settlements were more likely to deliver at home compared to their urban counterparts (Table 2).

## Discussion

Findings from our study suggest that home deliveries in Ghana have been decreasing in the past few years. The proportion of women who delivered at home were 50.56% in 2006, 31.35% in 2011, and 21.37% in 2017–2018. However, women who had less than eight ANC visits, dwelt in households with decreasing wealth, lived in the rural area, and resides in the Upper East region (in 2011 and 2017–18) were consistently at risk of delivering in the home throughout the three data waves. This suggest that the location of the woman and social status are significant factors in the choice of a place for delivery.

The home delivery prevalence reported in our study is consistent with that of 2015 Ghana Health Service statistics, and the findings of Ganle and colleagues (2019) who found that about a quarter of women still deliver at home in Ghana [34, 35]. The need for improvement in access to healthcare in Ghana is imperative. The Government of Ghana, like many governments of other developing countries, have recognized that delivery in unsafe and unsupervised conditions is common in the country [34]. The Therefore, over the years, the Government of Ghana has initiated several programs to improve maternal healthcare services including reduction of home deliveries through programs such as fee waiver for delivery in 2003, abolishment of delivery care cost in 2005, and the introduction of the National Health Insurance Scheme in 2005. Though these efforts have yielded some results, our results and other statistics revealed that home delivery is still an issue of great concern in Ghana [36]. Consistently, women from disadvantaged groups (such as rural, uneducated, households with lower socioeconomic status, and those from Upper East) had higher proportions of home deliveries compared to the national average. Women with no formal education or below secondary level education were more likely to deliver at home. This is because women with little or no education may not be adequately informed about the risks associated with home births [37, 38]. This is consistent with previous studies conducted in Ghana [5]. The government and development partners need to invest more in educational opportunities and expand the current Free Secondary School policy.

Results from this study suggest that though supervised deliveries are expected to be affordable and, in most cases, free in Ghana, several geographic, health system, and socio-demographic factors, prevent women from utilizing these services. From our study, ANC attendance was among the factors that were consistently associated with home deliveries across the years. The prevalence rate of home deliveries for women who attended ANC less than 4 times was almost three times as high as that of those who attended ANC 8 times or more. The finding is consistent with that of other studies done in Ghana [39, 40]. ANC has been reported to be a determinant of whether pregnant women will deliver within health facilities in Ghana [24]. Expectant mothers who are informed about pregnancy complications are more likely to deliver in healthcare facilities compared to uninformed pregnant women according to previous studies conducted in Tanzania and Bangladesh [41, 42]. Education during ANC helps allay fears or the perception women may have towards facility or supervised delivery. Supervised deliveries within health facilities provide women with information on the risks and complications they may encounter during labour and delivery. The World Health Organization’s decision to recommend eight ANC contacts instead of at least four contacts may have been influenced by these factors [43]. Risk assessment and medical examinations during ANC lead to early recognition of complications that may arise during and after delivery. Counselling and advice during ANC sessions on the need to seek supervised delivery positively influence women's decision to deliver within health facilities [44]. Other studies, however, have found that increased ANC visits may lead to a rise in the probability of home deliveries by expectant mothers [40].

Though the reduction in home deliveries over the past few years has been well documented, findings from our study and other studies [45] suggest that there are rural–urban differences. We found that 29.82% of women residing in rural areas delivered at home compared to 9.83% of women living in urban areas in 2017–2018. Though the percentage of rural women who delivered at home decreased from 2006 (65.29%) to 2018 (29.82%), it is still a far cry for home deliveries for urban women. This is consistent with the findings of studies from other African countries such as Nigeria [46], Tanzania [47], and Ethiopia [48]. In 2015, the Ghana Statistical Services reported a 59% versus 90% home deliveries in rural and urban areas in Ghana respectively [35]. The huge percentage of home deliveries among rural women is a major concern for the realization of the Sustainable Development Goals (SDGs) for reducing deaths among mothers and infants globally. The disparity in the number of health facilities in rural and urban areas in Ghana leads to a difference in accessibility to maternal health services, referrals, and specialist facilities. Also, the regions of the country with more rural areas especially the Upper East Region had greater prevalence rate of home deliveries compared to the more urban regions like Greater Accra region and Ashanti region. The regions with more rural areas have the most people with lower levels of education, low income, and beliefs that hinder them from accessing supervised deliveries. One of such beliefs is the perception that traditional birth attendants (TBAs) provided better care than the care given by skilled health professionals. This has been reported by various studies [41, 49, 50]. These findings underscore the relevance of improving collaboration between health facilities and TBAs as well as giving TBAs some form of training in delivery and referrals to reduce maternal mortality, especially in rural areas.

Household wealth and health insurance were factors that related to home deliveries according to our study results. Findings from our study and other studies suggest that home delivery decreased with an increase in financial stands [36, 51, 52]. The Government of Ghana has enacted policies such as the Community-based Health Planning and Services (CHPS) initiative nationwide in 2002, the free maternal health policy, and the National Health Insurance Scheme (NHIS) in a bid to improve access to maternal healthcare. Under the Ghana National Health Insurance Scheme policy, all pregnant women may enrol without paying the required premium. This has obviously improved access to maternal and childcare in Ghana. However, some women refuse to enrol in the scheme with the view that they may be made to pay additional money when they utilize supervised deliveries in health facilities. Previous studies have reported evidence of informal payments at the hospital despite enrolment in the NHIS [53, 54]. This trend worsens the already dire situation for poorer women without health insurance.

Based on our study findings we recommend that birth plan should include recognition of danger signs, a plan for place of delivery, and a plan for a skilled birth attendant. Also, efforts should be made to identify women who are not likely to receive skilled supervision in health facilities during ANC. Reasons for their potential refusal should be ascertained, and adequate support in terms of assistance with transportation to health facilities, follow-up, purposeful home visits, and counselling should be given to these women. Given the low prevalence rate of supervised deliveries in rural areas, efforts should be made to increase the number of health facilities, improve rural health services, enhance the quality of road networks linking urban and rural areas, and referral systems in rural areas. Also, to expand access to maternal health services in rural areas, telehealth and telemedicine can be utilized. Telehealth can take the form of remote patient monitoring, storage and transmission of medical information, and mobile health communication. The use of telehealth can reduce the burdens patients encounter such as traveling for specialty care. Telehealth can improve monitoring, communication, and timeliness of deliveries [55]. Barriers to access to supervised deliveries in rural areas can be addressed by creating awareness on negative beliefs and traditions that may influence maternal health. In addition, the free maternal health policy should be expanded to cover most medical supplies and services to reduce the financial burden on women and their families during supervised delivery. Although strengthening and encouraging enrolment in the NHIS will help improve supervised delivery, the management of various health facilities should address issues related to hidden costs and informal payments during supervised deliveries. Finally, access to secondary level education or higher needs to be improved by the government and development partners.

### Strengths and limitations of the study

A key strength of our study was the use of a large, nationally representative survey datasets collected in three waves by the Ghana Multiple Indicator Cluster Survey (GMICS) in 2006, 2011 and 2017/2018 based on a standardised methodology for analyses. Therefore, our findings can be generalized. Secondly, the study employed a complex sample analytic design to account for sampling units and weighting. Besides, the study unmasked the population of women who are at risk of home delivery, the associated sociodemographic factors and social inequalities as well as the progress made. The main limitation of the study is that we used secondary data which utilized a cross-sectional design. Hence, the associations observed in this study do not infer a causal relationship between the predictors and the outcome variables. The study was also restricted to variables available in the GMICS Data. Also, there was difficulty in determining the “where” and “how” of the previous child loss variable; it is not clear from the dataset or the questionnaire whether the experience of child loss occurred in a health facility or the home or any other place, therefore, it will be difficult to make any concrete conclusions on its effect on the place of subsequent delivery. From the summary statistics, however, it does appear that women who experienced previous child loss were associated with a higher likelihood of giving birth in the home in a bivariate model. Our recommendation for the designers of the GMICS questionnaire is that this question should have a follow-up question to ascertain where and how the respondent loss her child.

## Conclusion

Generally, the proportion of women who give birth at home has decreased. The proportion of home deliveries has reduced from 50.56% in 2006 to 21.37% in 2017–18. In the multivariable model, women who had less than eight antenatal care visits, dwelt in households with decreasing wealth, rural areas of residence, and residing in the Upper East region (in the year 2011 and 2017–18) were consistently at risk of delivering in the home throughout the three data waves. Policies should target the at-risk-women to achieve complete reduction in home deliveries. Access to facility-based deliveries should be expanded and ensure that the expansion measures are pro-poor, pro-rural, and pro-uneducated. Innovative measures such as mobile antenatal care programs can be organized in every community in the population segments that were consistently choosing home deliveries over facility-based deliveries.

## Data Availability

The datasets that were used in this study is freely available at https://mics.unicef.org/surveys once permission is sought and granted by UNICEF.
